# Effect of Sunflower and Marine Oils on Ruminal Microbiota, *In vitro* Fermentation and Digesta Fatty Acid Profile

**DOI:** 10.3389/fmicb.2017.01124

**Published:** 2017-06-20

**Authors:** Julio E. Vargas, Sonia Andrés, Timothy J. Snelling, Lorena López-Ferreras, David R. Yáñez-Ruíz, Carlos García-Estrada, Secundino López

**Affiliations:** ^1^Instituto de Ganadería de Montaña (CSIC-Universidad de León), Departamento de Producción Animal, Universidad de LeónLeón, Spain; ^2^Grupo CIENVET, Facultad de Ciencias Agropecuarias, Universidad de CaldasManizales, Colombia; ^3^Rowett Institute of Nutrition and Health, University of AberdeenAberdeen, United Kingdom; ^4^Department of Physiology/Metabolic Physiology, Institute of Neuroscience and Physiology, The Sahlgrenska Academy at the University of GothenburgGothenburg, Sweden; ^5^Estación Experimental del Zaidín, CSICGranada, Spain; ^6^INBIOTEC, Instituto de Biotecnología de LeónLeón, Spain

**Keywords:** microbial community composition, dietary fats, rumen microbiota, Rusitec fermenters, TRFLP, qPCR

## Abstract

This study using the rumen simulation technique (RUSITEC) investigated the changes in the ruminal microbiota and anaerobic fermentation in response to the addition of different lipid supplements to a ruminant diet. A basal diet with no oil added was the control, and the treatment diets were supplemented with sunflower oil (2%) only, or sunflower oil (2%) in combination with fish oil (1%) or algae oil (1%). Four fermentation units were used per treatment. RUSITEC fermenters were inoculated with rumen digesta. Substrate degradation, fermentation end-products (volatile fatty acids, lactate, gas, methane, and ammonia), and microbial protein synthesis were determined. Fatty acid profiles and microbial community composition were evaluated in digesta samples. Numbers of representative bacterial species and microbial groups were determined using qPCR. Microbial composition and diversity were based on T-RFLP spectra. The addition of oils had no effect on substrate degradation or microbial protein synthesis. Differences among diets in neutral detergent fiber degradation were not significant (*P* = 0.132), but the contrast comparing oil–supplemented diets with the control was significant (*P* = 0.039). Methane production was reduced (*P* < 0.05) with all oil supplements. Propionate production was increased when diets containing oil were fermented. Compared with the control, the addition of algae oil decreased the percentage C18:3 *c*9*c*12*c*15 in rumen digesta, and that of C18:2 *c*9*t*11 was increased when the control diet was supplemented with any oil. Marine oils decreased the hydrogenation of C18 unsaturated fatty acids. Microbial diversity was not affected by oil supplementation. Cluster analysis showed that diets with additional fish or algae oils formed a group separated from the sunflower oil diet. Supplementation with marine oils decreased the numbers of *Butyrivibrio* producers of stearic acid, and affected the numbers of protozoa, methanogens, *Selenomonas ruminantium* and *Streptococcus bovis*, but not total bacteria. In conclusion, there is a potential to manipulate the rumen fermentation and microbiota with the addition of sunflower, fish or algae oils to ruminant diets at appropriate concentrations. Specifically, supplementation of ruminant mixed rations with marine oils will reduce methane production, the acetate to propionate ratio and the fatty acid hydrogenation in the rumen.

## Introduction

Fats are added to the diets of ruminant livestock to increase energy density of the ration. Dietary oil supplements can also be used to manipulate the microbial community and fermentation processes in the rumen (Huws et al., 2010). Specific microbial groups and their interactions play a key role in several aspects of livestock production including environmental impact (Steinfeld et al., 2006), meat and milk quality (Shingfield et al., 2013), efficiency of feed utilization (Myer et al., 2015), health and welfare (Nagaraja and Titgemeyer, 2007). Therefore, the use of oil supplements to manage the microbiota and thereby promote measureable benefits in these aspects is of great interest.

Fats and oils impair ruminal CH_4_ production, and their inclusion in ruminant diets is considered by international agencies as one of the most feasible nutritional greenhouse gas (GHG) mitigation strategies (Bodas et al., 2012; Hristov et al., 2013). One of the global impacts on the environment from livestock agriculture is the release into the atmosphere of methane (CH_4_). Methane is a GHG with 21 times the global warming potential of CO_2_ (IPCC, 2007), and production from agricultural activity represents 10–12% of the total anthropogenic GHG equivalents per year (Gt CO_2_ Eq/yr) (Smith et al., 2014). Methane is produced in the rumen by the Archaea and these microbes are often found in close association with species of bacteria and ciliate protozoa, which produce H_2_ (Morgavi et al., 2010). However, beside the effects on methane emissions or methanogenic microbes, there is a need to demonstrate substrate degradability is not impaired (Hristov et al., 2013).

Oils from different sources may affect lipid metabolism in the rumen, and consequently the type of fatty acids absorbed in the intestine and subsequently present in the animal products. The fate of dietary oil supplements has been studied in depth and biohydrogenation pathways have been described in the reviews by Kim et al. (2009) and Lourenço et al. (2010). Milk and adipose fat obtained from ruminant livestock are sources of rumenic acid (C18:2 *c*9*t*11), an isomer of conjugated linoleic acid (CLA) that has been found to provide a number of benefits to human health (Bauman et al., 2005). *Butyrivibrio* spp. and related bacteria seem to be involved in producing CLA via the isomerization and biohydrogenation of polyunsaturated fatty acids (PUFA) in the rumen (Wallace et al., 2006; Lourenço et al., 2010). Recently, other reports have shown that other bacterial groups may be involved in biohydrogenation processes, although their contribution depends upon the lipid source since the toxic nature of the double bond may differ among different fatty acids (Huws et al., 2015). Rumenic acid is synthesized as an intermediate of the conversion of unsaturated linoleic acid to the saturated stearic acid. Therefore, the inclusion of supplements rich in PUFA in dietary rations, for example sunflower oil (ω-6-PUFA) or fish oil (ω-3-PUFA), could be used as an effective management practice to increase CLA in ruminant livestock products (Varadyova et al., 2008). Sunflower oil is rich in linoleic acid, the main precursor for the formation of CLA in the rumen. Fish and algae lipids are rich in long chain ω-3 PUFA (C20:5 ω-3 and C22:6 ω-3), and both have been considered suitable dietary supplements to provide essential long chain ω-3 PUFA and to increase their concentration in milk and meat (Korczyński et al., 2015). Fish oils or lipids contained in algae lessen the hydrogenation of fatty acids in the rumen then leading to the accumulation of unsaturated intermediates such as CLA (Wasowska et al., 2006). Thus, when diets are supplemented with fish or algae oils, dairy cows or sheep yield milk with higher contents in CLA (Shingfield et al., 2003). Supplementation of fattening lamb diets with algae oil increased C20:5 ω-3 and C22:6 ω-3 in meat to a greater extent than when the same diets were supplemented with fish oil (Cooper et al., 2004). Algae are increasingly used as a replacement for the commonly used fish oil (Stamey et al., 2012), reducing the problem of undesirable fishy smell and taste. In addition, algae are a valuable source of biological active, antioxidant and antimicrobial compounds (Korczyński et al., 2015). Technology for the cultivation of algae is rapidly developing given its productive (high yield of biomass), economic (production of food and feed, lipids, or biodiesel) and environmental (pollution prevention, wastewater treatment, detoxification of contaminants) benefits (Kouřimská et al., 2014; Wang et al., 2016).

When effects of dietary supplements have been investigated with the aim of manipulating the rumen microbiota to reduce environmental impact or improve the quality of the product, treatments should not adversely affect key species involved in the essential task of the breakdown of dietary fiber. *Butyrivibrio* spp. are important fiber-degrading bacteria (Maia et al., 2010). Prominent fiber-degraders (e.g., *Fibrobacter* and *Ruminococcus* spp.), and other dominant, nutritionally versatile groups (e.g., *Prevotella* spp.) should also be considered when a dietary supplement can potentially affect the composition of the rumen microbial community (Denman and McSweeney, 2006; Stevenson and Weimer, 2007). The balance of bacteria species is also responsible for the maintenance of a healthy ruminal pH. Therefore, it is of interest to examine if the addition of oils to the diet may affect lactate and volatile fatty acid (VFA) production and utilization.

There is a paucity of data measuring the effects of dietary oil supplements on other microbial species, particularly those with known roles in each of the several important aspects of rumen livestock agriculture described previously. Often, studies assessing the effects of lipid supplementation on rumen digestion do not address simultaneously impact on diet degradation, anaerobic fermentation, including methane production, microbial protein synthesis, lipid metabolism, and shifts in key microbial species and the whole community structure. Therefore, the aim of the present study was to investigate the effect of different types of marine oils used in combination with sunflower oil on rumen fermentation and the key microbial groups identified here. The experiment reported herein was carried out using an artificial rumen simulation technique (RUSITEC) (Czerkawski and Breckenridge, 1977), in which there is a precise control of the fermentation conditions and the aforementioned parameters can be measured in a single study.

## Materials and methods

Cultures of mixed ruminal microorganisms were maintained in semi-continuous flow fermenters (RUSITEC) to simulate rumen conditions. Sixteen fermentation vessels were set up using two RUSITEC systems. The addition of sunflower oil combined with either algae or fish oils to the basal diet was examined. Control (CTR) treatment was the basal diet (Table 1) with no addition of oil. The supplemented oil treatments were prepared by adding to the control diet either 20 g sunflower oil/kg diet alone (SFL) or combined with 10 g/kg of fish oil (FSH) or algae oil (ALG) to a total oil concentration of 30 g/kg (3%; Table 1). Diets used as fermentation substrate were prepared every 3 days and stored at 4°C until needed. Four vessels (two in each RUSITEC system) were used for each experimental treatment.

**Table 1:** Ingredients and chemical composition of control and oil supplemented diets.

**Treatment**	**Control**	**Sunflower oil**	**Sunflower and fish oil**	**Sunflower and marine algae**
**INGREDIENTS (g/kg DRY MATTER)**				
Cracked corn grain	250	245	243	243
Barley grain	150	147	146	146
Soybean meal	200	196	194	194
Lucerne hay	200	196	194	194
Beet pulp	100	98	97	97
Molasses	55	54	53	53
Sodium bicarbonate	15	15	15	15
Calcium carbonate	14	14	14	14
Dicalcium phosphate	6	6	6	6
Mineral vitamin premix	5	5	5	5
Marine salt	5	5	5	5
Sunflower oil	–	20	20	20
Fish oil	–	–	10	–
Algae marine oil	–	–	–	10
**COMPOSITION (g/kg DRY MATTER)**				
Organic matter	896	895	894	894
Crude protein	191	189	184	185
Neutral detergent fiber	354	390	364	368
Acid detergent fiber	139	145	141	142
Ether extract	16.0	37.2	47.3	48.9
**DIETARY FATTY ACID (FA) PROFILE (AS % OF TOTAL FA)**				
C14:0	1.09	0.60	1.30	1.78
C16:0	20.9	14.7	15.1	15.4
Total C18	76.3	82.8	72.2	66.7
C20:5ω3			1.35	0.27
C22:5ω6			0.28	3.73
C22:6ω3			4.05	8.85
C24:0				0.34
Otros	1.20	1.33	5.04	2.44
**DIETARY C18 FA (AS % OF TOTAL C18 FA)**				
C18:0	3.8	5.0	6.5	5.2
C18:1 *c*9	31.8	36.4	38.2	36.6
C18:2 *c*9*c*12	50.1	52.0	48.2	51.6
18:3 *c*9*c*12*c*15	14.3	6.7	7.2	6.6

*Fatty acid profile of oils (as % of total fatty acids)*.

*Sunflower oil: 8.7% C16:0, 5.3% C18:0, 35.6% C18:1ω9 c9, 47.3% C18:2ω6 c9c12, 0.3% C18:3ω3 c9c12c15*.

*Fish oil: 4.1% C14:0, 16.6% C16:0, 6.8% C18:0, 17.5% C18:1ω9 c9, 2.3% C18:2ω6 c9c12, 3.8% C18:3ω3 c9c12c15, 6.6% C20:5ω3 c5c8c11c14c17, 1.4% C22:5ω6 c4c7c10c13c16, 19.9% C22:6ω3 c4c7c10c13c16c19*.

*Algae oil: 6.4% C14:0, 18.4% C16:0, 1.0% C18:0, 1.8% C18:1ω9 c9, 0.5% C18:2ω6 c9c12, 0.1% C18:3ω3 c9c12c15, 1.7% C24:0, 1.3% C20:5ω3 c5c8c11c14c17, 18.3% C22:5ω6 c4c7c10c13c16, 43.5% C22:6ω3 c4c7c10c13c16c19*.

Vessels were inoculated and operated daily following the general incubation procedure of Czerkawski and Breckenridge (1977) and described in detail by García-González et al. (2010). To inoculate the fermentation units of RUSITEC, rumen digesta was obtained from four ruminally fistulated Merino sheep fed a diet consisting (per kg) of 700 g grass hay and 300 g concentrate. Sheep care and handling followed the recommendations of the Directive 2003/65 for protection of animals used for experimental and other specific purposes. The Animal Ethics Committees of CSIC and University of Leon (Spain) had approved procedures for digesta sampling from sheep. Inoculum was prepared from 400 ml of strained rumen fluid diluted with 250 ml of McDougall artificial saliva (McDougall, 1948). In addition, particulate rumen contents (80 g) were sealed inside a nylon bag (100 μm pore size), which was placed into the feed container of each fermentation vessel.

The dietary fermentation substrate was weighed (15 g dry matter) into a nylon bag, which was sealed and placed into the vessel feed container with the bag of strained rumen digesta. Incubation was started with temperature maintained at 37°C and a continuous infusion of McDougall artificial saliva (pH 8.4) at a rate of 556 ml/day (dilution rate of 3.6%/h) per vessel.

After the first 24 h of incubation, the bags with rumen contents were replaced by others with 15 g fermentation substrate. After 48 h the bag containing the original fermentation substrate was replaced. This procedure was repeated every 24 h replacing the bag containing the 48-h fermentation residue. Thus, every day in the feed container there was one bag with undigested residue after 48-h of incubation and another bag with a 24-h incubation residue.

### Experimental protocol: sampling and measurements

Sampling procedures were as described in detail by García-González et al. (2010). After a preliminary period of 6 days to reach steady state conditions, a measurement period started and lasted for 11 days (days 7–17). Samples to determine digestibility (as substrate disappearance from the bag) and fermentation end-products (VFA, lactate, gas, methane, and ammonia production) were collected on days 7, 8, 9, 14, 15, and 16 of the experiment.

Liquid effluent was collected in flasks to which 20 mL of 75 mM sulfuric acid had been added. Volume of effluent was measured daily and a sample (about 50 mL) was directly frozen (–18°C) for later ammonia, lactate and VFA analysis. Gas was collected in Tecator gas sampling bags (Tecobag, Tesseraux Container, Germany) and volume was measured by empting the bags with a vacuum pump connected to a flow meter (Ritter Apparatebau, Germany). A rubber septum was incorporated into the tubing for taking a sample using a syringe, and samples were kept in 10 mL vacuum tubes (Venoject®, Terumo Europe, Belgium).

On day 9, (^15^NH_4_)SO_4_ (95% enriched, Sigma, Madrid, Spain) was added into each vessel to dose 2.18 mg ^15^N to instantly label the NH_3_-N pool, and a solution of (^15^NH_4_)SO_4_ was added to the artificial saliva to reach a daily infusion rate of 4 μg/mg dietary N. The total effluent and the contents of the nylon bag collected from each vessel on day 12 were thoroughly mixed and homogenized in a blender and used to obtain a representative sample of total digesta and to isolate a microbial pellet following the procedures of Carro and Miller (1999). Non-ammonia N and ^15^N enrichment were determined in finely ground samples of total digesta and microbial pellets to estimate microbial N output.

On day 17, samples of digesta were collected from each vessel, and consisted of 30 mL of liquid content of the fermentation vessel, 1.5 g of fermentation residue from the 48-h bag, and 1.5 g of fermentation residue from the 24-h bag. The samples were mixed thoroughly, immediately frozen at –80°C and freeze-dried. Then, each sample was divided in two fractions. One portion was used for lipid extraction and fatty acid analysis. The other portion was homogenized by bead beating for 1 min (BioSpec Products) and used for DNA extraction using the QIAamp DNA Stool Mini Kit (Qiagen Inc., Valencia, CA, USA), according to the manufacturer's protocol with the exception of using a higher temperature (95°C) at the lysis step, required for cells that are difficult to lyse (such as some ruminal bacteria). DNA samples were used as templates for quantitative real-time polymerase chain reaction (qPCR) amplification and terminal restriction fragment length polymorphism (T-RFLP) analysis.

At the end of the experiment, the vessel liquid contents were used to inoculate batch cultures to determine fermentation gas production kinetics as indicator of the fermentative activity in each vessel.

### Chemical analyses

Proximate composition (moisture, ash, crude protein, ether extract, neutral, and acid detergent fiber) was determined using the analytical methods described by García-González et al. (2010). Methane in fermentation gas and VFA in effluent were determined by gas chromatography, ammonia nitrogen and lactate in effluent samples by colorimetry, and non-ammonia N and ^15^N enrichment in digesta and microbial pellets by isotope ratio mass spectrometry according to the methods described in detail by García-González et al. (2010). Fatty acid profile in samples of rumen digesta were determined according to Morán et al. (2013).

### T-RFLP and qPCR analysis

Total bacterial DNA was amplified by PCR using universal bacterial fluorescein-labeled oligonucleotide probes, forward primer 12f (5′-AGA GTT TGA TCC TGG CTC AG -3′) and reverse primer 1492r (5′-GGT TAC CTT GTT ACG ACT T-3′; Hongoh et al., 2003). The labeled PCR products were purified using GFX PCR DNA and Gel Band Purification kits (GE Healthcare, Madrid, Spain) and quantified. Each DNA sample (100 ng) was digested for 12 h at 37°C with the restriction enzymes *HhaI, HaeIII*, and *MspI*. The terminal restriction fragments (TRFs) were analyzed by capillary electrophoresis on an automatic sequence analyzer (MegaBACE 500, GE Healthcare) with internal Et-ROX labeled DNA size standards (Amersham Biosciences, GE Healthcare).

Finally, the allele report table generated by the Gene Marker program was used to calculate the relative height of each peak for each sample, then these data were analyzed by principal component analysis (PCA). The matrix of relative height of peaks detected from TRFs was used to calculate richness and diversity indices as described by Hill et al. (2003) and Lemos et al. (2011).

Quantitative real-time PCR was carried out using the Applied Biosystems StepOne Plus^*TM*^ Real Time PCR system (Applied Biosystems) using SYBR Green Supermix (Takara Bio Inc.) as described in Maeda et al. (2003). All reactions were performed in triplicate, using 10 μL of qPCR mix, 2 μL of DNA template, a variable volume (ranging between 0.5 and 2 μL, depending on the species or microbial group) of 10 μM solutions of each primer, and then adding PCR water up to a total volume of 20 μL per single reaction.

Primers were selected (Table 2) targeting the rRNA gene, specifically the 16S subunit for the bacteria and the 18S subunit for the ciliates and 18S and ITS1 for fungi. The methanogenic archaea were detected using primers targeting the methyl coenzyme-M reductase gene (*mcrA*) (Denman et al., 2007).

**Table 2:** Primers used for qPCR and T-RFLP.

**Primer**	**Sequence (5′–3′)**	**Target**	**References**
**qPCR**			
TotBacf	GTGSTGCAYGGYTGTCGTCA	Total Bacteria	Maeda et al., 2003
TotBacr	ACGTCRTCCMCACCTTCCTC		
qmcrA-F	TTCGGTGGATCDCARAGRGC	Methanogens	Denman et al., 2007
qmcra-R	GBARGTCGWAWCCGTAGAATCC		
316f	GCTTTCGWTGGTAGTGTATT	Ciliate protozoa	Sylvester et al., 2004
539r	CTTGCCCTCYAATCGTWCT		
fwd	GAGGAAGTAAAAGTCGTAACAAGGTTTC	Anaerobic fungi	Denman and McSweeney, 2006
rev	CAAATTCACAAAGGGTAGGATGATT		
PrF	GGTTCTGAGAGGAAGGTCCCC	*Prevotella* spp.	Stevenson and Weimer, 2007
PrR	TCCTGCACGCTACTTGGCTG		
FbsF	GGAGCGTAGGCGGAGATTCA	*Fibrobacter succinogenes*	Khafipour et al., 2009
FbsR	GCCTGCCCCTGAACTATCCA		
SrF	CAATAAGCATTCCGCCTGGG	*Selenomonas ruminantium*	Stevenson and Weimer, 2007
SrR	TTCACTCAATGTCAAGCCCTGG		
RaF	TGTTAACAGAGGGAAGCAAAGCA	*Ruminococcus albus*	Stevenson and Weimer, 2007
RaR	TGCAGCCTACAATCCGAACTAA		
MeF	GACCGAAACTGCGATGCTAGA	*Megasphaera* spp.	Ouwerkerk et al., 2002
MeR	CGCCTCAGCGTCAGTTGTC		
MeProbe	TCCAGAAAGCCGCTTTCGCCACT		Blanch et al., 2009
SbF	GATAGCTAATACCGCATAACAGCATT	*Streptococcus bovis*	Moya et al., 2009
SbR	AACGCAGGTCCATCTACTAGTGAA		
SbProbe	TGCTCCTTTCAAGCAT		
ScF	TGGGAAGCTACCTGATAGAG	*Succinivibrio* spp.	Tajima et al., 2001
ScR	CCTTCAGAGAGGTTCTCACT		
VAf	GCCTCAGCGTCAGTAATCG	*Butyrivibrio* VA (vaccenic acid producers)	Shingfield et al., 2012
VAr	GGAGCGTAGGCGTTTTAC		
SAf	TCCGGTGGTATGAGATGGGC	*Butyrivibrio* SA (stearic acid producers)	Paillard et al., 2007
Sar	GTCGCTGCATCAGAGTTTCCT		
MBP^*^	CCGCTTGGCCGTCCGACCTCTCAGTCCGAGCGG		
B395f	GYGAAGAAGTATTTCGGTAT	*Butyrivibrio* group	Boeckaert et al., 2008
B812r	CCAACACCTAGTA TTCATC		
**T-RFLP**			
27f	AGAGTTTGATCCTGGCTCAG	Universal bacteria	Hongoh et al., 2003
1389r	ACGGGCGGTGT GTACAAG		

* *Molecular beacon probe*.

Quantitative PCR analysis of the *Butyrivibrio* SA-producing bacteria [or *B. proteoclasticus* group including the species causing a complete hydrogenation of unsaturated 18 C fatty acids to stearic acid (SA)] was carried out using the molecular beacon approach with the primers and probe designed by Paillard et al. (2007) (Table 2).

Standards used for copy number calculations were prepared using a plasmid vector containing the respective PCR amplicon sequence of each of the microbial groups. The use of plasmids as PCR standards are described in detail by Andrés et al. (2016). The identity of the standard amplicons were verified by sequencing the plasmid insert and confirming sequence identity using BLAST (http://www.ncbi.nlm.nih.gov/BLAST/).

### Fermentative activity

On the last experimental day, batch cultures were inoculated with the liquid contents of the vessels for studying the kinetics of gas production in the different inocula upon specific incubation of different substrates. The substrates used were cellulose (Sigmacell®, Sigma-Aldrich Química SA, Madrid, Spain), corn starch (Calbiochem®, Merck, Germany), and the same mixed substrate used in the Rusitec vessels (CTR diet, Table 1), ground to 1 mm. On day 17, bags of substrate were withdrawn and liquid was kept in the vessels, which were closed and left at 39°C for 4 h. Meanwhile, 500 mg of DM of the substrates were weighed into 120 mL serum bottles. Then, the fluid of each RUSITEC vessel was anaerobically dispensed into each serum bottle (50 mL each), inoculating two bottles per substrate and two blank bottles (without any substrate), which were sealed and crimped, and then incubated at 39°C for 92 h. No additional buffer was added to the bottles. Gas production was measured using a pressure transducer (Theodorou et al., 1994) at 3, 6, 9, 12, 16, 20, 24, 29, 34, 44, 56, 68, 80, and 92 h. Nonlinear regression was performed using PROC NLIN of SAS to fit the exponential model of France et al. (2000) to cumulative gas production data:

(1) $$G=A\left[1-{\text{e}}^{-k_{\text{d}}(t-L)}\right],$$

where *G* is cumulative gas production (mL/g) at incubation time *t* (h), *A* is the asymptotic gas production (mL/h), *k*_*d*_ is the fractional fermentation rate (h^−1^), and *L* is the lag time (h). From this equation, estimations were obtained for gas produced at 24 h (*G*24, mL/g), and average gas production rate (*R*, mL/g h^−1^) between inoculation and time at which gas production was equal to half of *A* (France et al., 2000).

### Statistical analysis

Measurements taken on several days were averaged for each vessel. Data were analyzed using the SAS program (SAS Institute Inc. 2011. SAS/STAT® 9.3 User's Guide. Cary, NC: SAS Institute Inc.) with ANOVA using a randomized complete block design. Within each experiment, treatments were randomly assigned to two vessels of each RUSITEC system, thus giving four replicates per treatment. Each vessel (fermentation unit) was an experimental unit, and the RUSITEC system was the blocking factor. The statistical model was:

(2) $$y_{ijk}=\mu +B_{i}+O_{j}+\varepsilon_{ijk}$$

where *y*_*ijk*_ is the value for an individual observation, μ the overall mean, *B*_*i*_ the effect of the blocking factor (*i* = RUSITEC system 1 or 2), *O*_*j*_ is the fixed effect of oil treatment (*j* = 1…4; CTR, SFL, FSH, or ALG), and ε_*ijk*_ is the residual error. The random effect was fermentation vessel *k* within treatment (*j*) and block (*i*). Statistical significance was declared at *P* < 0.05 and *P* < 0.10 was considered a tendency. Multiple comparisons of means among oil sources were performed using Tukey's test.

Similarity and grouping of treatments based on T-RFLP spectra were assessed using cluster and principal components analyses.

## Results

There were no significant differences (*P* > 0.05) in ruminal pH between diets, although average pH was significantly decreased (*P* = 0.027) when the diet was supplemented with an oil. The addition of oils had no apparent effect on substrate degradation (assessed by measuring disappearance from the nylon bags containing the incubated diet), except for fat digestibility, which was higher (*P* < 0.001) when oil was added to the diet. Furthermore, fat digestibility was increased (*P* = 0.010) when a marine oil was supplemented compared with the diet containing only sunflower oil. There were no significant differences among the four diets in neutral detergent fiber (NDF) digestibility (*P* = 0.132), but the contrasting comparison of the CTR diet with average of all treatments supplemented with an oil (SFL, FSH, and ALG diets) reached the level of statistical significance (*P* = 0.039). Methane production was consistently reduced (*P* < 0.05) when oil supplements were added to the diet (Table 3). Total VFA production was increased when diets containing supplementary oil were fermented, mainly as result of the increase in propionate and valerate production (Table 3). Propionate production was significantly increased (*P* < 0.05) when diets supplemented with marine oils were fermented compared with the control, resulting in a reduced acetate to propionate ratio (Table 3). Lactate production was also increased in diets with marine oils compared with the control diet. The additions of oils to the diet did not affect ammonia or microbial protein synthesis.

**Table 3:** Effects of oils added to the diet on ruminal fermentation in RUSITEC fermenters.

**Item**	**Control (CTR)**	**Sunflower oil (SFL)**	**SFL + Fish oil**	**SFL + Marine algae**	**SEM (*n* = 4)**	***P*-value**	***P*-value contrast CTR vs. OIL**	***P*-value contrast SFL vs. MARINE**
Effluent, mL/d	594	585	628	591	19.9	0.458	0.786	0.346
pH	6.78	6.73	6.74	6.72	0.015	0.100	0.027	0.892
**DISAPPEARANCE COEFFICIENTS, g DIGESTED/100 g INCUBATED**								
Dry matter	73.3	73.0	73.1	73.7	1.05	0.958	0.973	0.768
Organic matter	72.7	72.2	72.3	73.1	1.10	0.926	0.939	0.705
Neutral detergent fiber	53.5	57.2	56.2	55.3	1.00	0.132	0.039	0.340
Acid detergent fiber	27.9	28.6	30.8	28.9	1.51	0.596	0.415	0.526
Crude protein	70.6	72.3	71.2	72.8	1.50	0.754	0.455	0.863
Ether extract	65.4^b^	81.5^a^	85.0^a^	88.9^a^	2.43	0.002	<0.001	0.010
**FERMENTATION GAS PRODUCTION**								
Total gas, L/d	3.03	2.95	3.05	2.91	0.086	0.658	0.583	0.784
CH_4_, L/d	0.265^a^	0.222^ab^	0.207^b^	0.211^b^	0.0101	0.008	<0.001	0.355
CH_4_, mL/100 mL total gas	8.98^a^	7.86^b^	7.54^b^	7.23^b^	0.226	0.002	<0.001	0.124
CH4, mmol/g FOM	1.20^a^	1.01^ab^	0.94^b^	0.94^b^	0.050	0.011	0.002	0.334
**VOLATILE FATTY ACID (VFA, mmol/d)**								
Acetate	25.5	27.4	26.5	26.7	0.49	0.175	0.061	0.257
Propionate	6.94^c^	7.38^b^^c^	8.36^ab^	8.46^a^	0.176	<0.001	<0.001	0.002
Butyrate	8.30	8.87	8.81	8.51	0.210	0.297	0.144	0.433
Valerate	2.56^c^	2.88^b^	3.19^a^	3.04^ab^	0.053	<0.001	<0.001	0.006
Isoacids	2.24	2.29	2.46	2.41	0.063	0.075	0.060	0.070
Total VFA	47.8^b^	51.3^a^	51.4^a^	51.3^a^	0.83	0.055	0.008	0.966
Acetate:propionate ratio	3.72^a^	3.54^ab^	3.19^b^	3.18^b^	0.099	0.012	0.009	0.014
L-lactate, mg/d	9.1^c^	10.1^b^^c^	14.0^a^	13.5^ab^	0.81	0.003	0.004	0.004
Ammonia N, mg/d	171	156	171	165	4.2	0.140	0.211	0.062
Microbial protein, g/d	1.39	1.35	1.41	1.37	0.072	0.924	0.894	0.638
Microbial protein, g/100 g FOM	14.1	13.7	14.3	13.7	0.75	0.919	0.842	0.728

*CTR, Control diet; SFL, control diet supplemented with 2% sunflower oil; SFL + fish oil, control diet supplemented with 2% sunflower oil + 1% fish oil; SFL + algae oil, control diet supplemented with 2% sunflower oil + 1% algae oil*.

*SEM, Standard error of the mean*.

*Contrast CTR vs. OIL: comparison between CTR and all treatments supplemented with oil*.

*Contrast SFL vs. MARINE: comparison between SFL and treatments supplemented with marine oils*.

*FOM, Fermentable organic matter*.

a,b *Within a row, mean values without common superscript letters differ (P < 0.05)*.

The addition of oil to the diet gave rise to variations in the digesta fatty acid profile, changing the proportions of C14:0 (greater with ALG than with CTR or SFL diets), C16:0 (lower with SFL and FSH than with CTR diet), total C18 (greater with SFL than with ALG diets), and C20 and C22 PUFA (only present in treatments supplemented with marine oils; Table 4). Compared with CTR diet, the addition of algae decreased the percentage of C18:3 *c*9*c*12*c*15 in rumen digesta. The percentage of C18:2 *c*9*t*11 was increased and that of C18:2 *t*10*c*12 was decreased when the control diet was supplemented with an oil (Table 4). Ruminal biohydrogenation of C18 fatty acids was calculated according to Wu and Palmquist (1991). Compared to the CTR diet, the addition of sunflower and fish oils (FSH treatment) reduced the hydrogenation of C18 PUFA (C18:2 *c*9*c*12 and C18:3 *c*9*c*12*c*15) and increased the hydrogenation of C18:1 *c*9. Biohydrogenation of C18:3 *c*9*c*12*c*15 was greater with FSH than with ALG diets, and that of C18:2 *c*9*c*12 was higher with FSH than with any of the other diets tested. The addition of fish oil or algae with sunflower oil to the CTR diet resulted in a decrease of the overall hydrogenation of C18 unsaturated fatty acids. There were no differences among treatments supplemented with oils (SFL, FSH, or ALG diets) in total C18 fatty acid biohydrogenation.

**Table 4:** Effects of oils added to the diet on ruminal digesta fatty acid (FA) profile and C18 fatty acid biohydrogenation in RUSITEC fermenters.

**Item**	**Control (CTR)**	**Sunflower oil (SFL)**	**SFL + Fish oil**	**SFL + Marine algae**	**SEM (*n* = 4)**	***P*-value**	***P*-value contrast CTR vs. OIL**	***P*-value contrast SFL vs. MARINE**
**DIGESTA FATTY ACIDS (AS % of TOTAL FA)**								
C14:0	2.01^b^	1.92^b^	2.53^ab^	3.01^a^	0.160	0.011	0.091	0.008
C14:1	1.41	1.34	1.16	1.35	0.165	0.940	0.622	0.840
C15:0	1.59	1.17	1.50	1.38	0.163	0.350	0.256	0.204
C15:1	0.439	0.300	0.452	0.318	0.059	0.210	0.250	0.288
C16:0	22.8^a^	17.8^b^	18.2^b^	20.1^ab^	0.88	0.012	0.003	0.241
C16:1	4.09^a^	3.83^ab^	4.59^a^	3.19^b^	0.185	0.006	0.281	0.965
Total C18 FA	62.0^ab^	66.5^a^	64.1^ab^	59.1^b^	1.53	0.041	0.535	0.029
C20:5n3	0.0^b^	0.0^b^	0.50^a^	0.42^a^	0.117	0.017	0.045	0.008
C22:6n3	0.0^b^	0.0^b^	1.32^a^	1.59^a^	0.198	<0.001	0.002	<0.001
Others	6.29	6.18	7.09	6.48	0.788	0.854	0.733	0.562
**C18 FA PROFILE (AS % of TOTAL C18 FA)**								
C18:0	17.5	15.1	13.4	14.4	1.45	0.390	0.126	0.550
C18:1 *t*11	2.23	2.33	1.87	2.76	0.674	0.874	0.903	0.992
C18:1 *c*9	29.5	29.6	27.6	31.6	1.47	0.340	0.907	0.975
C18:1 *c*11	4.63	3.47	4.49	3.04	0.449	0.077	0.088	0.611
C18:2 *c*9*c*12	31.2	40.6	40.3	40.8	2.95	0.139	0.028	0.716
C18:3 *c*6*c*9*c*12	0.30	0.25	0.34	0.33	0.040	0.401	0.944	0.102
C18:3 *c*9*c*12*c*15	5.62^a^	4.36^ab^	5.25^ab^	4.08^b^	0.328	0.038	0.024	0.513
C18:2 *c*9*t*11	0.049^b^	0.275^a^	0.337^a^	0.343^a^	0.029	<0.001	<0.001	0.105
C18:2 *t*10*c*12	0.391^a^	0.276^b^	0.298^ab^	0.291^ab^	0.022	0.043	0.008	0.623
Others	2.18	2.00	1.81	1.74	0.198	0.472	0.205	0.421
**BIOHYDROGENATION %**								
C18:3 *c*9*c*12*c*15	52.7^a^	34.4^ab^	27.1^b^	38.3^a^	3.91	0.012	0.004	0.733
C18:2 *c*9*c*12	34.7^a^	29.8^a^	8.5^b^	24.7^a^	2.96	0.009	0.019	0.024
C18:1 *c*9	6.5^b^	21.2^ab^	27.7^a^	13.5^ab^	4.17	0.040	0.028	0.917
Total C18 biohydrogenation	27.3^a^	21.3^ab^	17.3^b^	18.9^b^	1.89	0.027	0.006	0.260

*CTR, Control diet; SFL, control diet supplemented with 2% sunflower oil; SFL + fish oil, control diet supplemented with 2% sunflower oil + 1% fish oil; SFL + algae oil, control diet supplemented with 2% sunflower oil + 1% algae oil*.

*SEM, Standard error of the mean*.

*Contrast CTR vs. OIL: comparison between CTR and all treatments supplemented with oil*.

*Contrast SFL vs. MARINE: comparison between SFL and treatments supplemented with marine oils*.

a,b *Within a row, mean values without common superscript letters differ (P < 0.05)*.

There were no differences among inocula from RUSITEC vessels fed the different diets in the fermentation of the control diet or cellulose (Table 5). In contrast, fermentation kinetics of starch showed that the addition of fish or algae oils had a slight inhibitory effect on microbial amylolytic activity in the rumen (Table 5).

**Table 5:** Effects of oils added to the diet on fermentative activity in RUSITEC fermenters.

**Item**	**Control (CTR)**	**Sunflower oil (SFL)**	**SFL + Fish oil**	**SFL + Marine algae**	**SEM (*n* = 4)**	***P*-value**	***P*-value contrast CTR vs. OIL**	***P*-value contrast SFL vs. MARINE**
**CONTROL DIET**								
Asymptotic gas production, mL/g	321	321	312	328	9.3	0.676	0.903	0.914
Fractional fermentation rate, h^−1^	0.050	0.049	0.055	0.051	0.0024	0.324	0.695	0.205
Lag time, h	1.3	1.4	1.7	1.6	0.26	0.456	0.179	0.429
Average fermentation rate, mL/h	10.6	10.4	10.9	10.6	0.46	0.820	0.764	0.458
Gas production at 24 h, mL/g	217	215	220	221	7.1	0.936	0.955	0.537
**STARCH**								
Asymptotic gas production, mL/g	414	419	400	422	15.8	0.780	0.980	0.704
Fractional fermentation rate, h^−1^	0.111	0.107	0.104	0.096	0.0054	0.270	0.184	0.264
Lag time, h	2.2^b^	3.0^ab^	3.5^ab^	5.1^a^	0.51	0.012	0.016	0.061
Average fermentation rate, mL/h	24.7^a^	22.2^ab^	20.1^b^	18.1^b^	1.04	0.006	0.003	0.035
Gas production at 24 h, mL/g	377	374	347	346	11.1	0.132	0.120	0.067
**CELLULOSE**								
Asymptotic gas production, mL/g	388	384	356	382	23.3	0.760	0.627	0.604
Fractional fermentation rate, h^−1^	0.046	0.048	0.051	0.047	0.044	0.924	0.661	0.886
Lag time, h	14.9	15.3	16.6	16.0	1.53	0.859	0.534	0.619
Average fermentation rate, mL/h	6.6	6.4	5.9	6.1	0.40	0.603	0.345	0.374
Gas production at 24 h, mL/g	122	122	100	112	16.2	0.753	0.590	0.432

*CTR, Control diet; SFL, control diet supplemented with 2% sunflower oil; SFL + fish oil, control diet supplemented with 2% sunflower oil + 1% fish oil; SFL + algae oil, control diet supplemented with 2% sunflower oil + 1% algae oil*.

*SEM, Standard error of the mean*.

*Contrast CTR vs. OIL: comparison between CTR and all treatments supplemented with oil*.

*Contrast SFL vs. MARINE: comparison between SFL and treatments supplemented with marine oils*.

a,b *Within a row, mean values without common superscript letters differ (P < 0.05)*.

**Figure 1:**
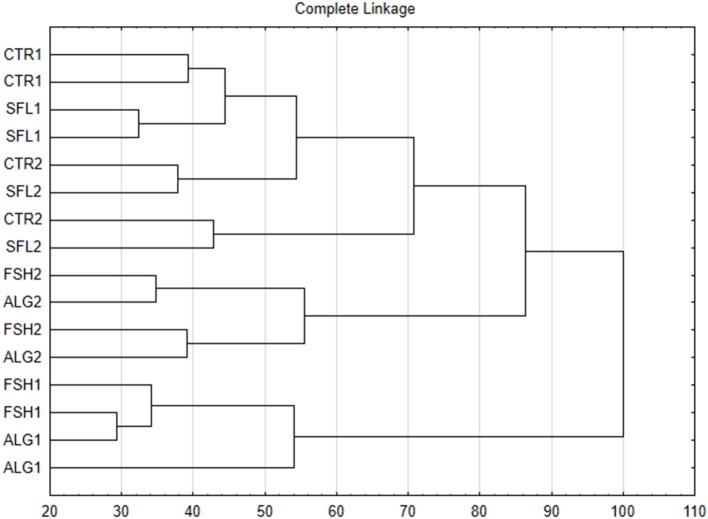
Dendrogram derived from the analysis of T-RFLP data showing the grouping of RUSITEC bacterial communities provided with either control diets (CTR) or diets supplemented with 2% sunflower oil (SFL) oil, sunflower oil with 1% fish oil (FSH) or sunflower oil and 1% marine algae (ALG). The number after the treatment code indicates the RUSITEC system (there were two RUSITEC systems and two replicates of each treatment in each system).

**Figure 2:**
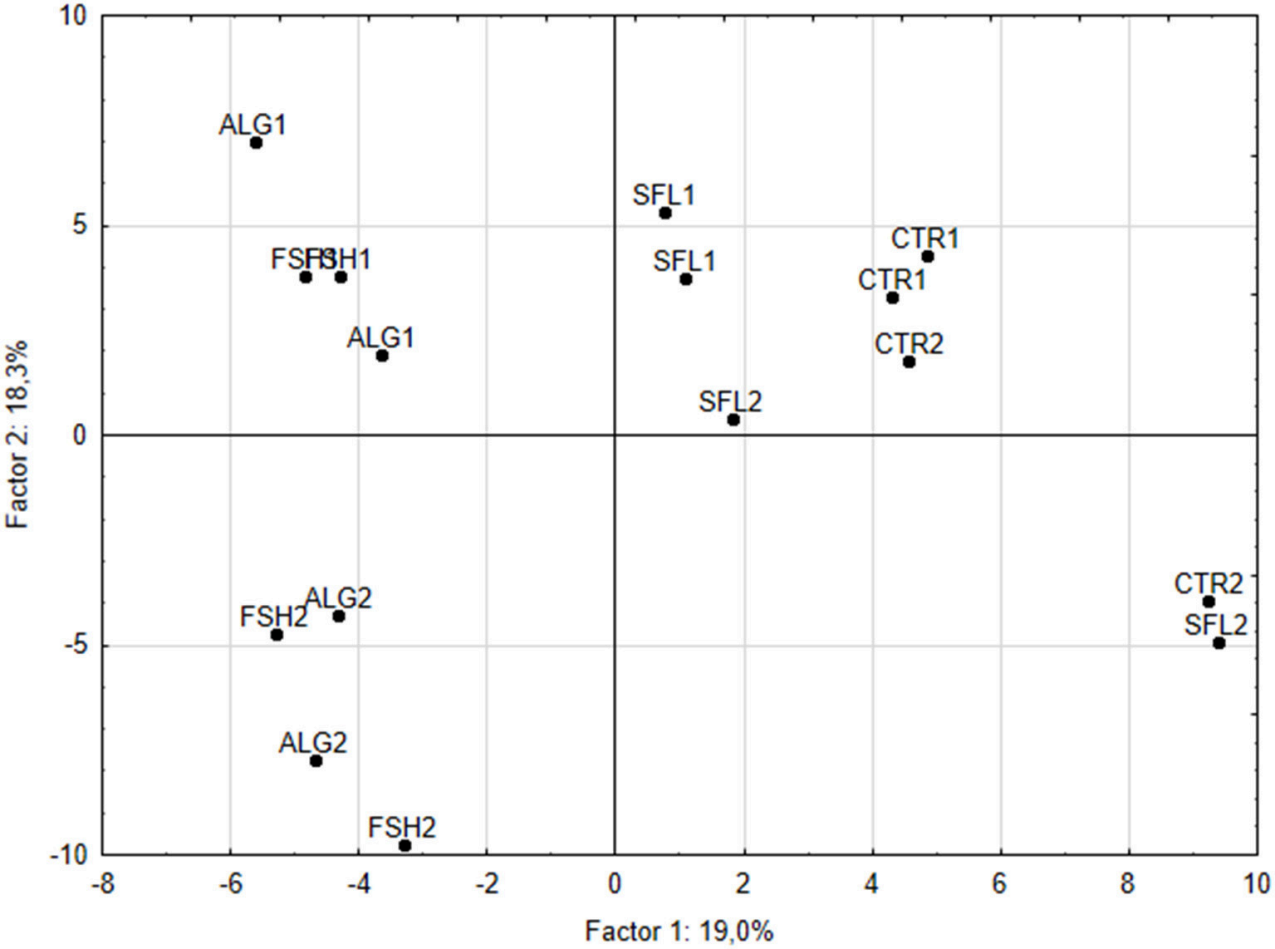
Principal components plot derived from the analysis of T-RFLP data showing the discrimination along principal components 1 and 2 of RUSITEC bacterial communities provided with either control diets (CTR) or diets supplemented with 2% sunflower oil (SFL) oil, sunflower oil with 1% fish oil (FSH) or sunflower oil and 1% marine algae (ALG). The number after the treatment code indicates the RUSITEC system (there were two RUSITEC systems and two replicates of each treatment in each system).

**Figure 3:**
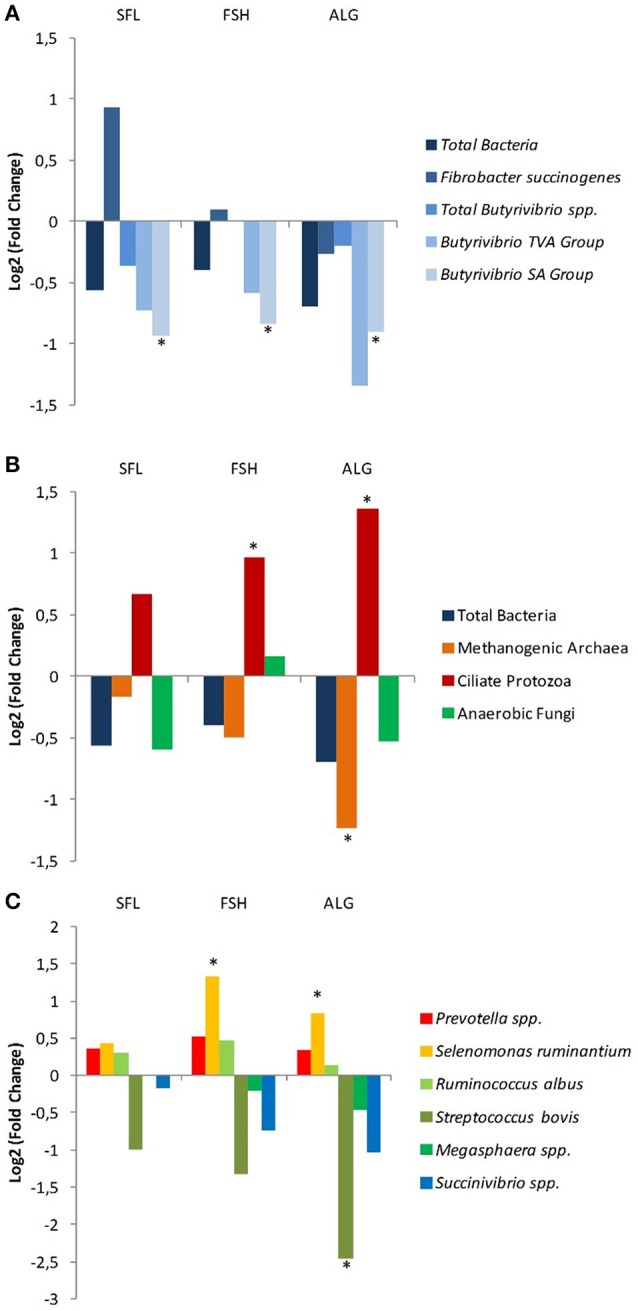
Relative quantitation compared to control diet of 16S rRNA copy numbers of rumen microbiota groups after supplementation of the control diet with sunflower oil (SFL) or with combinations of SFL and fish oil (FSH) or marine algae (ALG). Fold-changes for specific amplicon groups were calculated as the (log 2) ratio of normalized abundances. **(A)** Total bacteria and microbial groups involved in biohydrogenation. **(B)** Major prokaryotic and eukaryotic domains found in the rumen including the Archaeal groups responsible for the production of ruminal methane. **(C)** Other key bacteria groups including the dominant *Prevotella* spp. and species involved in fiber degradation, and lactate metabolism. ^*^Significant difference in copy number (*P* < 0.05) compared with the control.

Both cluster analysis (Figure 1) and PCA (Figure 2) showed clear discrimination between microbial profiles in the vessels fed CTR or SFL diets and those receiving the FSH or ALG diets. Treatments were clearly separated along the factor 1 (explaining up to 19% of the variance), indicating that this principal component would reflect the effects of dietary oil addition on microbial composition. The fragments with a greatest contribution to this principal component were identified and the differences between treatments in these fragments were examined. A theoretical assignment of the more eccentric TRFs to compatible bacterial species was performed using the tap-tRFLP tool of the Ribosomal Data Project (Cole et al., 2003). Based on this analysis it would seem that supplementation with FSH and ALG would increase bacteria belonging to genera *Fibrobacter* and *Prevotella*, and would reduce abundance of bacteria of *Butyrivibrio, Ruminococcus, Proteobacteria, Actinobacteria, Lachnospira*, and *Streptococcus* genera. The supplementation of the control diet with oils did not significantly affect measurements of species richness or diversity (Table 6) or total bacterial numbers (Figure 3A). The specific microbial groups affected by the addition of oils are shown in Figures 3A–C. The addition of sunflower oil alone had no effect (*P* > 0.05) on any of the microbial groups examined. This effect on the *Butyrivibrio* SA numbers was also seen when the diet was further supplemented with fish or algae oils (Figure 3A). The diets supplemented with fish and algae oils increased the numbers of ciliate protozoa and reduced the counts of methanogenic archaea (Figure 3B), although the decreased in methanogens was only significant with the ALG diet. Diets FSH and ALG increased the abundance of *Selenomonas ruminantium*, and ALG diet also decreased the presence of *Streptococcus bovis* (Figure 3C). No other significant diet effects were observed on any of the other microbial groups quantified by qPCR.

**Table 6:** Diversity indices of the RUSITEC bacteria community provided with control diets, or diets containing sunflower oil only, or sunflower oil with algae or fish oil supplementation.

**Index**	**Control (CTR)**	**Sunflower oil (SFL)**	**SFL + Fish oil**	**SFL + Marine algae**	**SEM (*n* = 4)**	***P*-value**	***P*-value contrast CTR vs. OIL**
Species richness, S	84.8	80.5	78.3	65.8	8.73	0.418	0.320
Shannon's diversity index, H'	3.78	3.73	3.64	3.49	0.128	0.280	0.250
Shannon's evenness index, E	0.855	0.849	0.836	0.848	0.016	0.834	0.484
Simpson's diversity index, D	0.043	0.050	0.056	0.061	0.0092	0.397	0.222
Index of diversity, 1-D	0.957	0.950	0.944	0.939	0.0092	0.397	0.214
Number of species accounting for 50% total relative peak height	6.3	5.5	5.0	4.0	1.54	0.608	0.375
Number of species accounting for 70% total relative peak height	14.0	13.5	12.0	10.5	2.65	0.596	0.440

*CTR, Control diet; SFL, control diet supplemented with 2% sunflower oil; SFL + fish oil, control diet supplemented with 2% sunflower oil + 1% fish oil; SFL + algae oil, control diet supplemented with 2% sunflower oil + 1% algae oil*.

*SEM, standard error of the mean*.

*Contrast CTR vs. OIL: comparison between CTR and all treatments supplemented with oil*.

## Discussion

If supplementation of dietary oils in ruminant livestock diets can provide even small improvements in terms of more efficient production, better health and welfare of the animal or reduction of environmental impact, then there are clear benefits to their use. This study measured the changes in rumen fermentation and microbial community composition, in particular in prominent microbial groups associated with key aspects of ruminant livestock production, in response to the supplementation of the diet with different combinations of sunflower and fish oil as well as a marine algae based product. Microbial communities were examined in digesta samples obtained from fermenters in which ruminal fermentation is simulated *in vitro*, characterized by a precise control of inputs (feed and saliva) to and outputs (fermentation end-products) from the system, and of the fermentation conditions (e.g., pH, temperature). The use of live animals was avoided by maintaining the microbial communities in a simulated rumen environment.

The diversity of the total bacterial community was measured using T-RFLP. Richness and diversity of species were not affected by the supplementation with SFL either with or without the addition of the fish oil and marine algae. In both the clustering and principal component analyses, the RUSITEC vessels formed groups separate from the effects of the treatment. This effect was considered a normal effect of the variability in the microbial community between systems. For instance, in the case of the vessels receiving only SFL (2%), the treatment effect was not as strong as the system effect. However, the samples containing the additional 1% fish oils or algae formed a group separated from the SFL only and control treatments. A similar discrimination of bacteria community composition has been reported in rumen fluid obtained from sheep supplemented with different combinations of 2% sunflower and/or 1% fish oil (Belenguer et al., 2010). Significant effects on the bacterial community have been detected when grass or red clover silage diets were supplemented with 0, 1, 2, or 3% fish oil, but with a minor effect on richness of species (Huws et al., 2010). Diets supplemented only with 2.5% sunflower oil, or with this plant oil plus incremental amounts (0.8, 1.6, or 2.4%) of marine algae were clustered according to the ruminal bacterial composition (Toral et al., 2012) in a comparable grouping as in our study.

Analysis by qPCR of the individual species and microbial groups proved a more sensitive measurement of the effects of the different oils. The addition of 2% SFL with or without additional 1% FSH and ALG caused a non-significant fold reduction in the copy number of total bacteria relative to the control. This result was broadly reflected in the results measuring the total microbial protein synthesis (g/d). An inhibitory effect of oils on rumen bacteria has been reported in a number of studies *in vivo* (Vargas-Bello-Pérez et al., 2016) and *in vitro* pure cultures (Maia et al., 2010) although in these cases, a higher dose may have contributed to a greater effect.

General bacterial diversity and abundance measured by T-RFLP agreed with the rumen fermentation results. Substrate degradation was not affected and total VFA production was increased by the addition of oils to the diet. The addition of fat to ruminant diets has been previously associated with a reduction of feed digestibility, in particular of fiber degradation (Palmquist and Jenkins, 1980). This effect has been attributed to changes in the microbial communities, a reduction in fermentative activity or the limitation of the access of microbes and enzymes to feed particles due to physical coating (Nagaraja et al., 1997). In our study, bacterial groups involved in fiber degradation were not substantially modified. *Ruminococcus albus* showed a small increase in copy number, and the dominant ruminal fibrolytic bacteria *Fibrobacter succinogenes* was not significantly affected by oil supplementation. Hence, the ability to breakdown dietary fiber in the rumen was not negatively affected by the addition of oils at the doses used in these experiments (Denman and McSweeney, 2006). In concordance with these observation, the fermentative activity on pure substrates (cellulose or starch) assessed by gas production kinetics was not affected when the different fat supplements studied were added to the control diet. The amount and type of lipid added (Jenkins, 1993) may explain the discrepancy of the effects of oil addition on bacteria numbers and substrate degradation observed in our study with others reported in the literature. With levels of added oils similar to that used in our study, Doreau et al. (2009) concluded that organic matter fermentation in the rumen was not affected. In a recent meta-analysis, Weld and Armentano (2017) showed that the inclusion of fats to dairy cow diets did not affect total tract and ruminal fiber digestibility. Patra (2014) concluded that fiber digestibility was only adversely affected when fat was added to sheep diets at high concentrations. Also in agreement with our study, Gao et al. (2016) observed an increase in VFA concentration in batch cultures of mixed ruminal microorganisms fed with substrates supplemented with different oil sources. Differences among diets in NDF degradation were not significant (*P* = 0.132), but the contrast comparing the control vs. oil-supplemented diets became significant (*P* = 0.039). This finding was unexpected, as only few studies have observed an effect similar to that seen in our study (e.g., Kim et al., 2008). It is noteworthy that none of the diets supplemented with an oil was significantly different from the control diet, and that whereas the contrast was significant for NDF, it was not so for acid detergent fiber. On the other hand, cellulolytic activity as determined with a highly sensitive method (gas production fermenting cellulose in the incubation medium from Rusitec fermenters), total VFA or acetate production and abundance of fibrolytic bacteria (*F. succinogenes* and *R. albus*) were not different among treatments, indicating that effects of oil supplementation on NDF digestibility would be rather subtle. Overall, considering all the data comprehensively it would be sound to assume that regardless the statistical significance for that particular contrast, the effect of oil supplementation on NDF degradation is of little biological relevance.

Copy numbers of *Succinovibrio* spp. as an example of the rumen Proteobacteria group was reduced. However, there were few exceptions with members of the bacteroidetes *Prevotella* spp. and *S. ruminantium* apparently benefitting from the addition of the oil and algae supplements. These nutritionally versatile groups may have been able to expand their niche as a result of the general reduction in numbers of other species.

Ciliate protozoa numbers were significantly increased with the addition of 1% FSH and ALG to the 2% SFL, and those of methanogens significantly reduced, particularly after ALG supplementation. The significant increase of ciliate protozoa in the present study was unexpected. Lipids have been reported as potential defaunating agents, albeit at much higher doses (Newbold and Chamberlain, 1988). Previous *in vivo* studies reported ciliate numbers were either unaffected (Boeckaert et al., 2008) or reduced (Boeckaert et al., 2007) following the addition of an algae supplement in the diet. However, the treatments excluded the addition of SFL and could merely highlight the differences between ciliate growth *in vivo* and *in vitro*. Martínez et al. (2010) reported protozoa numbers in RUSITEC fermenters were orders of magnitude reduced from and not representative of the protozoal population observed in rumen of sheep fed a similar diet.

A reduction of the number of methanogens caused by dietary oil supplementation was associated with the reduced rate of ruminal methane production by the RUSITEC fermenters. Methanogenesis has been reported to be correlated with methanogen numbers (Wallace et al., 2015). In our study, whereas the production of total fermentation gas was not affected by the inclusion of oils in the diet, the methane concentration in the gas was reduced by 16% on average compared with that observed with the CTR diet. As a result, methane production (expressed either as volume per day or as mmol/g fermented OM) was significantly reduced when FSH or ALG were added to the diet. Similar oil based dietary supplements have been found to reduce methane production *in vitro* (Castagnino et al., 2015). The link between dietary fat supplementation and a reduction in ruminal methane production has been also shown by compiling data from different studies (Eugène et al., 2008). Another meta-analysis showed that fats added at low concentration to sheep diets may decrease methane production without affecting rumen fermentation (Patra, 2014), in agreement with our results in RUSITEC fermenters. Methane reduction by oil supplementation concurred with changes in the VFA profile, in particular with an increase in propionate production and a decreased acetate to propionate ratio. The relative increase in propionate production in response to the inclusion of dietary fats has been observed *in vitro* (Potu et al., 2011) and *in vivo* (Shingfield et al., 2012).

Ruminal biohydrogenation of PUFA has been well-documented (Jenkins et al., 2008). Linoleic acid is converted to CLA (C18:2 *c*9*t*11), which is then hydrogenated to vaccenic acid (C18:1 *t*11). Linolenic acid is converted to C18:3 *c*9*t*11*c*15 and C18:2 *t*11*c*15 as intermediates and finally hydrogenated to vaccenic acid. Under the highly reducing conditions prevailing in the rumen, vaccenic acid may be further saturated to stearic acid (C18:0). Less is known about the biohydrogenation of ω-3 PUFA having more than three double bonds (C20 or C22). It has been observed that these fatty acids disappear rapidly when incubated in cultures of mixed ruminal microorganisms (AbuGhazaleh and Jenkins, 2004), probably by isomerization, hydrogenation and chain shortening leading to the formation of *trans*-C18:1, although the intermediate steps have not been elucidated.

Anaerobic fungi have been found to play a role in the biohydrogenation of linoleic and linolenic acids (Nam and Garnsworthy, 2007). The addition of the different oil and algae supplements had a variable non-significant effect of the copy numbers of fungi relative to the control. Therefore, it is possible that other microorganisms had a more relevant contribution to biohydrogenation of fatty acids in our RUSITEC fermenters. The *Butyrivibrio* bacteria groups include species that are associated with the biohydrogenation of unsaturated fatty acids (Wallace et al., 2006). The toxicity of unsaturated acids on isolates of species within this group of bacteria has already been reported (Maia et al., 2010) as well as the reduction of copy number of *Butyrivibrio* spp. *in vivo* following the supplementation of vegetable oils (Kim et al., 2008) or algae (Boeckaert et al., 2008). It is apparent that there is considerable animal-to-animal variation in the response of rumen microbes to dietary oil (Belenguer et al., 2010). In the RUSITEC, oil or algae supplementation consistently reduced copy number of *Butyrivibrio* spp. relative to control, the stearic acid producing group (SA) being significantly affected by the SFL, FSH, and ALG treatments. In our study, total biohydrogenation of C18 fatty acids was reduced with the addition of fish oil or algae. The addition of dietary fish oil at 1% or 3% of dry matter intake has been found to affect adversely some bacterial species, in particular *Butyrivibrio* spp., and to reduce the biohydrogenation of fatty acids in the rumen (Kim et al., 2008). Stearic acid in the ruminal digesta was reduced in all the oil supplemented treatments, although the differences with the control diet did not reach statistical significance. Rumenic acid (C18:2 *c*9*t*11) was significantly increased in all oil supplemented treatments, probably in response to the sunflower oil, as no further increase was observed when fish oil or algae were added to this vegetable oil. The effects of oil supplementation on fatty acid profile and biohydrogenation in ruminal digesta depend on the type and amount of oil added and on the diet to which the oil is incorporated (Shingfield et al., 2012). The addition of vegetable or marine oils at an appropriate dose could, in theory, contribute to the production of healthier products from ruminant livestock, provided this is not accompanied by an overall decrease in fat synthesis (Shingfield et al., 2006).

Copy numbers of *S. bovis* were reduced with the addition of the SFL, FSH, and ALG supplements, significantly in the latter. As a prominent lactate producer, *S. bovis* is associated with acidosis in livestock ruminants often as a response to the feeding of high concentrate diets (Petri et al., 2013). By reducing the proliferation of this microbial group with the addition of oils or algae to the diet it may be possible to remediate such a condition. However, the production of lactate was significantly increased with the addition of FSH or ALG to the diet possibly as a result of a proliferation of other lactate producing species such as the *Lactobaccilli* not measured here. Despite the increase in lactate, it was not sufficient to significantly reduce the rumen pH. Moreover, the relative copy number of the corresponding lactate consumer *Megasphaera elsdenii* was not affected by the incorporation of oils to the diet. Studies with lambs (Ferreira et al., 2016) or dairy cows (Shingfield et al., 2003) reported a slight increase in rumen pH when feed was supplemented with fish oil. This effect was not observed in our *in vitro* study where the incubation medium in the fermenters is strongly buffered, so variations in pH may be not representative of those occurring *in vivo*. Lipid supplements increase the energy concentration of the diet without increasing the risk of rumen acidosis, but further research on the effects of adding oils to ruminant diets on lactate production and utilization is warranted.

Using the RUSITEC system it was demonstrated that there can be real potential to manipulate the rumen microbiota with the addition of vegetable and fish oils and marine algae supplements. The balance of the various groups of the microbial community can in turn contribute to a number of important aspects of livestock agriculture described previously. The effect on the microbial community of the supplementation was reflected in VFA and methane production activity with a significant shift toward propionate production and lower emissions. Moreover, there were concomitant changes in long chain fatty acid profiles and in biohydrogenation of unsaturated fatty acids without adversely affecting ruminal fermentation. Therefore, our findings support and add to the results of previous studies linking dietary fish oil supplements and the fatty acid composition in milk (Shingfield et al., 2006; Toral et al., 2012) and methane production (Eugène et al., 2008).

## Author contributions

SL and SA conceived of and designed the experiments. JV and SL (Rusitec experiments, sampling), SA (TRFLP), LL (qPCR), and CG (DNA analysis) performed the experiments. SL, SA, JV, TS, and DY analyzed the data. The manuscript was written by TS, SA, and SL, and all authors contributed to refining the text and approved the version to be submitted.

### Conflict of interest statement

The authors declare that the research was conducted in the absence of any commercial or financial relationships that could be construed as a potential conflict of interest.
